# Stages of change of behavior in women on a multi-professional program for treatment of obesity**^1^**

**DOI:** 10.1590/1518-8345.0549.2809

**Published:** 2016-10-10

**Authors:** Cheila Aparecida Bevilaqua, Sandra Marisa Pelloso, Sonia Silva Marcon

**Affiliations:** 2 MSc, Assistant Professor, Campos Videira, Instituto Federal Catarinense, Ciência e Tecnologia, Videira, SC, Brazil.; 3 PhD, Full Profesor, Departamento de Enfermagem, Universidade Estadual de Maringá, Maringá, PR, Brazil.

**Keywords:** Obesity, Women, Feeding Behavior, Motor Activity

## Abstract

**Objective:**

to ascertain the effectiveness of an intervention program in relation to anthropometric measurements and stage of readiness for behavioral change in women with excess weight.

**Methods:**

the intervention group (IG) was made up of 13 women, and the control group (CG), by 20. The intervention lasted 16 weeks, and included the practice of guided physical activity three times a week, and health education once a week. The application of the questionnaire on stage of readiness for behavioral change, and the anthropometric evaluations, were undertaken at two points - before and after the period of intervention. The statistical analysis involved tests of comparison and association.

**Results:**

in general, at the first point, the participants in the two groups were predisposed to make changes in what they ate and in their physical activity. However, significant difference was only observed in relation to weight, body mass index (BMI), waist circumference and waist-hip ratio and readiness for change among the members of the intervention group.

**Conclusion:**

the intervention programmed was effective in weight loss, reduction of waist circumference and waist-hip ratio, and in changing behaviors related to the practicing of physical exercise and eating habits.

## Introduction

The increase in the prevalence of obesity found in various countries characterizes this situation as an epidemic and a worldwide problem^1^. Since the 1980s, the rates of obesity have increased by about three times, in regions such as the Middle East and countries such as China and Australia. Even in countries with a lower prevalence, the rates observed are considered high. It is estimated that, in the coming two decades, the increase in the number of new cases of obesity will be over hundreds of millions^2^.

It is possible to observe that in countries where obesity affects a large proportion of the population, such as the United States, investments are made in public and private initiatives with the aim of preventing obesity among the young through Special Nutrition Programs, interventions, programs and actions encouraging healthy eating and the undertaking of physical activity^1^ ^-^ ^3^.

It is known that genetic and metabolic factors directly influence weight gain; however, other factors, such as physical inactivity, an unhealthy diet and psychosocial stress increase the risk of developing obesity. As a result, interventions geared towards these factors must be undertaken to prevent or reverse the situation of obesity in the individual and populational ambit^4^.

In spite of obesity and the proposals for its treatment being widely publicized, as far as is known, there have been few intervention studies aiming for the control not only of body mass, but principally changes in behaviors of risk. In this regard, as part of the therapeutic process, it is important to assess and determine how the individual feels in relation to a possible change in behavior, and how to motivate the person to change his or her lifestyle, as this will be reflected directly in the results desired and in healthier standards^5^. 

As a result, the Stages of Readiness for Behavioral Change (SRBC) model, or trans-theoretical model (TM), is used for assessing behaviors related to the practice of physical activity and eating and to propose necessary intervention strategies^6^ ^-^ ^7^. It allows one to classify the individuals in their respective stages of change, making a distinction between those subjects who are genuinely disposed to change their lifestyle, and those who do not have the intention of doing so. It is emphasized that the changes tend to occur more efficiently among those who are disposed to change behaviors^6^.

In the light of the above, this study's objective was to ascertain the effectiveness of an intervention program on the anthropometric variables and on the Stage of Readiness for Behavioral Change among women with excess weight. 

## Method

An intervention study, of the before and after type, undertaken with adult women with excess weight (BMI≥25 kg/m²), resident in the municipality of Paiçandu in the Brazilian state of Paraná (PR).

The sample was made up of women recruited in a study titled "Populational study regarding the prevalence of risk factors and protection factors for cardiovascular diseases in the metropolitan region of Maringá (PR)", undertaken in Maringá, Paiçandu and Sarandi. In the municipality of Paiçandu, a total of 415 individuals were evaluated, of whom 287 were female. Of the latter, 83 were overweight. All were invited to participate in this study. 

The 51 women who accepted to participate in the study were randomly allocated to two groups, 25 in the intervention group (IG), and 26 in the control group (CG). Only 33 were evaluated at the end of the 16 weeks of intervention: 13 in the IG, and 20 in the CG. The six women who dropped out of the CG did not attend for assessment at the second point, in spite of numerous attempts at contact. In the IG, 12 women were excluded for various reasons, with one having an orthopedic fracture, two becoming pregnant, and nine alleging difficulty in traveling to the place where the interventions were undertaken and/or lack of time. 

All 33 women participating were subjected to a general protocol of assessment including: identification of the sociodemographic characteristics, the taking of a patient history, aptitude for the practicing of physical exercise, anthropometry and Stage of Readiness for Behavioral Change (SRBC). The anthropometric assessments were undertaken through measurements of body mass, height, and circumference of the waist and hips. Height was determined with the person standing straight, their arms extended by their side and with the hands turned towards the thighs. The feet were bare, with the heels together and the weight equally distributed through both feet. 

The measurement of the waist circumference (WC) was taken using a flexible tape measure, at the midpoint between the costal margin and the iliac crest, with the individual standing up and breathing normally. Central obesity was considered to be present when WC was superior to 88 cm. In addition, the measurements referent to body mass index (BMI) and waist-hip ratio were made using the Biospace InBody 520^(r)^ multifrequency bioimpedence device, which as well as being available in the signatory institution, allows the study participants themselves to see the distribution of their body mass. 

The ability for the practicing of physical exercises was determined through the application of the PAR-Q questionnaire (Physical Activity Readiness Questionnaire)^8^.

The evaluation of the food-related SRBC and of physical activity (PA) was made through applying the Stage of change (SoC) questionnaire, based on the transtheoretical model proposed by American researchers and validated for the Brazilian context^9^. The SRBC is determined according to the mean score obtained; values between 1 and 1.4 indicate a stage of precontemplation; between 1.5 and 2.4, contemplation; between 2.5 and 3.4, preparation; between 3.5 and 4.4, action; and between 4.5 and 5, maintenance^10^. In order to ascertain the global stage of readiness for behavioral change, the general mean of the scores obtained in each one of the domains was ascertained. 

The intervention program was undertaken in the hall of the church located in the center of the city, over 16 weeks. The assessments undertaken in the pre- and post-intervention points took place outside this period in the two groups. All the materials, apparatus and equipment (mattresses measuring 1.20 x 0.60 meters, stethoscope, manual sphygmomanometer, heart rate monitor and portable digital weighing scales, with a maximum capacity of 150 kg and an accuracy of 0.1 kg) used during the intervention or in the assessments belonged to the researchers. It is emphasized that during the undertaking of the activities, alternative equipment was used, such as broom handles for stretching and 500 mL plastic bottles containing sand for lifting weights using the upper limbs.

Body mass (in kilograms) was determined using a portable digital weighing scales (maximum capacity of 150 kg, and accuracy of 0.1 kg). For determining height (in meters), a circumference measuring tape was used. For calculating Body Mass Index (BMI), the individual's weight (in kilograms) was divided by the square of the person's height (in meters). The values were classified in: normal weight BMI >18.50 to 24.99 kg/m^2^, overweight BMI ≥25 to 29.99 kg/m^2^ and obesity BMI ≥30 kg/m^2(^ ^9^. Central obesity (concentration of adipose tissue in the abdominal region) was determined when the abdominal circumference was superior to 102 cm for men and 88 cm for women^9^, measured at the midpoint between the costal margin and the iliac crest. 

The intervention included the practicing of physical exercises, nutritional guidance, and education in health. The physical exercises were undertaken three times a week, for 60 minutes, structured in three points: initial warm-up and stretching (15 min), aerobic activity followed by localized exercises (35 min) and final stretching (10 min). The participants were monitored using heart rate monitors, with the specific intensity for each activity determined through the maximum cardiac frequency obtained through the one mile walk test^11^.

Health education and nutritional guidance took place fortnightly on an alternative basis, over 60 minutes, and were always held on Mondays, prior to the program of exercise. The health education was undertaken by a nurse, with sporadic participation from specialists in the areas of endocrinology, nephrology, speech therapy, occupational therapy and pharmacy, who provided specific guidance related to their areas of work. 

The nutritional guidance was provided in the group, without individual diet-related prescriptions. This involved various issues such as the food pyramid, energy density and the nutritional composition of foods, the importance of macro- and micronutrients, the appropriate way of preparing and consuming these, and encouragement to create new food-related behaviors.

Prior to undertaking the comparisons of the results obtained in the pre- and post-intervention points, the extent was ascertained to which the individuals who had dropped out of the study resembled those who participated in it until the end; it was observed that there was no significant difference between these in relation to the variables of body and biochemical composition (lipid profile). Once evidenced that the loss was random and not intentional, the results of the intervention were compared. It is highlighted that it was not investigated whether the members of the control group began/undertook a diet or physical activity during the study period. 

The SPSS statistical package, version 18.0, was used for the statistical analysis of the data; and the Shapiro-Wilk test was used for the distribution of these data. The descriptive statistics used for characterization of the sample involve the measurements of central tendency and dispersion (mean, median, standard deviation and interquartile range). In the comparisons between the pre- and post-intervention points, in relation to the domains/questions, the Student's t-test was used for the dependent samples, and Wilcoxon's nonparametric test for the dependent samples. The improvement in the anthropometric variables was ascertained based on the difference between the final and initial measurements. The Chi-squared test and Fisher's exact test were used to check the association between the reduction of the anthropometric variables and the general SRBC in the pre-intervention phase. A level of significance of 5% was adopted in all the analyses. 

The undertaking of the study was approved by the Standing Committee for Ethics in Research with Human Beings (COPEP) of the State University of Maringá (Opinion N. 546/2011) and the wider project from which this study derived was approved by the Brazilian Clinical Trials Registry (RBR-6673s5). All the participants signed two copies of the terms of free and informed consent. 

## Results

The mean age of the women in the IG was 43.08 (±11.79) years old, and in the CG was 49 (±8.86) years old. In relation to the purchasing power, the majority of the women were classified as being from class C (70% and 75% of the IG and CG, respectively), while the other women belonged to class A/B. In the IG, four women were overweight, seven had grade I obesity and two had grade II obesity. In the CG, four were overweight, 12 had grade I obesity, two had grade II obesity, and two had grade III obesity. 

After 16 weeks, the women from the IG presented significant reduction for all the variables analyzed. In the CG, no reduction was observed; on the contrary, an increase occurred in the mean of three of the four measurements analyzed (Table 1). 

**Table 1:** Comparison of the means of the anthropometric measurements of the two groups at the pre- and post-intervention points. Paiçandu, State of Paraná (PR), Brazil, 2012

Variables	Intervention Group (n=13)				Control Group (n=20)		
	Pre-	Post-	p		Pre-	Post-	p
Weight*	79.59(±11.86)	77.22(±11.35)	0.004		75.00(±11.25)	76.01(±10.96)	0.235
BMI^†‡^	31.95(±4.03)	28.95(±9.47)	0.003		31.42(±3.76)	32.14(±4.12)	0.111
WC*^§^	93.54(±8.20)	89.23(±8.25)	<0.001		96.95(±8.24)	97.75(±8.21)	0.145
WHR^||‡^	0.980(±0.043)	0.970(±0.049)	0.002		1.000(±0.043)	1.000(±0.047)	0.104

*Student's t-test; †BMI: Body Mass Index; ‡Wilcoxon's test; §WC: Waist circumference; ||WHR: Waist-hip ratio.

It is emphasized that in spite of the CG having presented, for some variables, a higher mean value after the weeks of the study, no evidence was found through the hypothesis test that there had been change between the two evaluations in this group. 

It is underlined that of the 20 women in the CG, 15 were in the preparation stage and of these, five remained in this stage until the end, while the others regressed to the precontemplation stage. In relation to the four women who were in the contemplation stage, three remained at this stage and one regressed to the precontemplation stage; the only woman who was in the action stage regressed to the preparation stage.

Considering only the members of the IG, one can observe in Table 2 that prior to the intervention, three were in the contemplation stage. Of these, one progressed to the preparation stage, and two to the action stage. The three who were in the preparation stage (3) progressed to the action stage. However, of the seven women who were in the action stage (4), five regressed to the preparation stage. Regardless of these results, at the end of the intervention period, the majority of them (12) presented reduction of the variables of weight, BMI, WC and WHR.

**Table 2:** Global Stage of Readiness for Behavioral Change (SRBC) and percentage difference of the anthropometric measurements in women from the Intervention Group (IG) before and after the intervention. Paiçandu, PR, Brazil, 2012

n	Age (years)	Global SRBC*			Weight	BMI^†^	WC^‡^	WHR^§^
		Pre-	Post-		Percentage Difference			
1	51	4	3		-6.42	-7.0	-2.27	-4.16
2	25	3	4		0.60	0.7	-9.30	0
3	43	4	3		-4.50	-4.9	-5.88	-1.03
4	36	2	3		-5.32	-5.8	-5.10	-1.98
5	51	2	4		-1.75	-1.8	2.38	-1.01
6	28	2	4		-0.73	-0.8	-3.84	-0.97
7	55	4	4		-1.15	-1.2	-1.86	-0.98
8	59	4	3		-5.08	-5.0	-3.40	-0.97
9	46	4	3		-0.41	-0.7	-8.51	-1
10	57	4	3		-3.04	-3.0	-3.33	-0.97
11	31	3	4		-3.91	-4.0	-10.46	-2.24
12	30	3	4		-4.96	-5.3	-6.79	-2.04
13	48	4	4		0.40	0.3	-1.16	-1.01

*SRBC: Stage of Readiness for Behavioral Change; †BMI: Body Mass Index; ‡WC: Waist circumference; §WHR: Waist-hip ratio.

It is emphasized that among the participants from the CG, reduction was not observed in the means of any of the anthropometric variables studied. Of the 20 women in this group, 15 were in the stage of preparation, and of these, five remained thus through to the end, while the others regressed to the stage of precontemplation. In relation to the four women who were in the contemplation stage, three remained in this stage, and one regressed to the precontemplation stage; the only woman who was in the action stage regressed to the preparation stage. 

Table 3 presents the frequencies obtained by the women in both groups in each one of the domains of the SRBC at the pre- and post-intervention points. Positive changes were observed in the women of the IG, represented by the progression between the stages in the four domains, an aspect which was not observed among the women of the CG. 

**Table 3:** Distribution of the study participants, at the pre- and post-intervention points, in relation to the domains of the Stage of Readiness for Behavioral Change (SRBC). Paiçandu, PR, Brazil, 2012

Domains		Pre-Contemplation		Contemplation		Preparation		Action		Maintenance	
		Pre-	Post-	Pre-	Post-	Pre-	Post-	Pre-	Post-	Pre-	Post-
Intervention Group (n=13)											
	1 - Size and quantity of the portions*	-	-	3	-	4	2	6	11	-	-
	2 - Quantity of fat in the diet^††^	-	-	1	-	5	1	6	12	1	-
	3 - Consumption of fruits and greens*	-	-	1	1	8	6	4	6	-	-
	4 - Physical activity Control Group*	-	-	3	-	2	2	4	4	4	7
Control (n=20)											
	1 - Size and quantity of the portions*	2	1	9	12	6	5	2	2	1	-
	2 - Quantity of fat in the diet^†^	1	-	5	3	11	15	2	2	1	-
	3 - Consumption of fruits and greens*	-	6	11	5	6	6	3	3	-	-
	4 - Physical activity*	1	-	-	5	6	8	10	7	3	-

*Wilcoxon test for paired samples; †Student's t-test for paired samples

Regarding the behavior of what people eat, one can observe, in Table 4, significant differences between the two points in relation to six behaviors in the IG and to two in the CG, it being the case that in the IG, the changes were related to the size of the portions and quantity of foods ingested, and also to the adoption of a diet low in fat, although no other behavior which characterizes this type of diet underwent a change. Attention is also called the absence of change in relation to the consumption of fruits and vegetables. Among the women of the CG, significant change was observed only in relation to the dressing used in the preparation of salads, and in the substitution of fried foods by vegetables. 

**Table 4:** Comparison between the pre- and post-intervention points, in relation to the domains of the Stage of Readiness for Behavioral Change (SRBC) - eating-related behaviors in each group, Paiçandu - PR, Brazil, 2012

Domains		Group		
		Intervention	Control	
		p	p	
Domain 1 - Size and quantity of the portions				
	I limit the quantity that I eat and do not eat more than I need.	0.023*	0.160	
	I measure and weigh the portions of the foods that I eat.	0.002*	0.450	
	I eat less in later meals if I exaggerated in previous meals.	0.410	1.000	
	I stop eating before I feel "full - stuffed".	0.050	0.788	
	I avoid eating when I'm nervous, sad or depressed.	0.152	0.269	
	I drink a glass of water before meals.	0.014*	0.880	
	I resist eating everything on the plate if I'm no longer hungry.	0.526	0.344	
	I "keep track" of how much I am eating when I am snacking.	0.023*	0.732	
	I say no to second portions.	0.234*	0.190	
Domain 2 - Quantity of fat in the diet				
	I eat a diet low in fats.	0.020*	0.225	
	I eat chicken without the skin.	0.837	0.577	
	I drink skimmed milk and eat low-fat dairy products (yogurt, cheese).	0.568	0.497	
	I remove all the fat from poultry.	0.193	0.577	
	I limit the size of the portions of meat in meals.	0.053	0.236	
	I avoid fried foods such as French fries, chicken or polenta.	0.291	0.313	
	I avoid fast food (hamburgers, French fries etc.).	0.433	0.101	
	I avoid snacks such as chips, peanuts or popcorn.	0.570	0.415	
	I have stopped putting butter and/or margarine on bread, crackers and cakes.	0.082	0.091	
	I use low-fat dressing for salad	0.213	0.022*	
	I avoid cake, cookies and pies.	0.075	0.081	
Domain 3 - consumption of fruits and greens				
	I eat at least 5 portions of fruits and vegetables a day.	0.084	0.074	
	I eat at least 3 portions of green vegetables (broccoli, spinach) a day.	0.904	0.888	
	When I order food, I request vegetables instead of French fries.	0.063	0.004*	
	I eat at least two portions of fruit every day.	0.218	0.811	
	I eat green salads and vegetables such as carrots and tomatoes.	0.589	0.042	
	I include fruits with my meals, such as bananas with cereals or papaya.	0.839	0.577	
	I eat fruits as puddings.	0.135	0.396	
	I include vegetables with my meals, such as lettuce or tomato in sandwiches.	0.347	0.915	
	When I snack, I snack on fruits.	0.250	0.715	

* significant value for p<0.05

One can observe in Table 5 that the women of the IG presented significant difference in the three aspects related to physical activity and which denote the inclusion of some daily attitudes, such as adopting a routine with various activities, parking the car some distance away so as to walk more, and undertaking heavy work at work. The women of the CG, on the other hand, presented significant difference in relation to two aspects: the use of the stairs instead of the elevator, and parking the car a certain distance away.

**Table 5:** Comparison in the pre- and post-intervention points, in relation to the domain of the Stage of Readiness for Behavioral Change (SRBC) of physical activity, in each group. Paiçandu, PR, Brazil, 2012

Domains		Group	
		Intervention	Control
		*p*	*p*
Domain 4 - in relation to physical activity			
	I include a variety of physical activities in my daily routine.	0.018*	0.191
	I spend a good part of my time away from my table, doing more active tasks.	0.112	0.323
	I do heavy cleaning, such as cleaning windows and scrubbing the floor and walls.	0.066	1.000
	I do heavy work at work, I lift objects or use heavy machinery.	0.011*	0.157
	I do gardening, clean the backyard and paved areas.	0.194	0.100
	I seek ways to be active in my daily routine, I don't use the TV remote control, don't use the cordless telephone, and wash up by hand.	0.666	0.077
	I do active things at the end of the day, such as walks to visit friends or to stroll.	0.062	0.534
	I use the stairs instead of the lift or the escalator.	0.172	0.023*
	I park the car some distance from where I have to go, and walk to the place.	0.027*	0.025*

* Significant value for p<0.05

## Discussion

For the anthropometric indicators, a significant reduction was observed in the mean values for body weight, BMI, WC and WHR only among the women of the IG, reinforcing the efficacy of the intervention (physical activity and nutritional advice). The literature indicates that interventions related to practicing physical activity and nutritional guidance with women are capable of promoting changes in the anthropometric parameters, mainly in those related to body mass and WC^12^ ^-^ ^14^.

The inclusion of new habits in the routine is indispensable for the success of any intervention, especially when one compares various conditions prior to its beginning and after its end, but also such that behaviors resulting from the intervention, and triggers of positive results, may be maintained in the long-term. As a result, assessing the readiness for behavioral change is important, as it determines the real possibilities of this occurring, to the extent that it allows one to distinguish individuals who are disposed to changing their lifestyle from those who do not have well-defined plans in relation to this. In this way, one can avoid a negative evaluation of the intervention in the cases in which the unsatisfactory results may be related more to the fact of the individual not wanting to change behaviors/habits. 

In the present study, it was identified that in the beginning, three participants of the IG were in the contemplation stage, three planned to begin actions in the near future, and seven had already begun them. In the CG, four women were in the contemplation stage, 15 in the preparation stage, and one in the action stage. Generally speaking, therefore, the proportion of women who were in the preparation stage and action stage may be considered similar in the two groups (76.9% in the IG and 80.0% in the CG). This constitutes a positive aspect, as what is indicated most in intervention studies is that participants are in these stages, as they indicate that the same are disposed to practicing new behaviors^10^.

In spite of the two groups' similarity, reduction in the means of the anthropometric measures was only observed in the IG, which shows the importance of the intervention, characterized by the practicing of physical activity and nutritional guidance for women with excess weight. Another aspect which deserves to be highlighted is the fact that five women who presented regression in the stage of readiness for action presented reduction in the values of the anthropometric variables, which may have resulted from the practicing of physical activity proposed by the intervention. 

One study undertaken in Brazil with 662 adolescents indicated the direct relationship between the stages of behavioral change and motivation for practicing physical exercise. It was emphasized that as individuals advance in the stages of behavioral change, they increase their strength of motivation for exercise and this makes the subjects more self-determined to include physical exercise as a daily routine^15^ ^).^.

It is highlighted that the simple progression from one stage of the transtheoretical model to another more advanced stage allows the individual to present a profile which is more favorable to physical activity and/or eating habits, thus reducing the risks to his or her health^16^.

In this regard, it was observed that the IG presented significant changes in the stages of readiness for change after the 16 weeks of the intervention. This indicates that the advice related to appropriate foods, and to the regular practicing of physical exercise, caused the IG participants to effectively include certain changes in their day-to-day. These changes involve three domains of the SRBC: the size and quantity of the portions, the quantity of fat in the diet, and the practice of physical activity. The control group did not present changes in any of the domains. 

One study undertaken with 90 Iranian women showed there to exist a significant association between the loss of body weight and the SRBC, emphasizing that for effective changes to occur it is necessary for the individual genuinely to perceive her health condition and to be in a stage of preparation and/or action. It also indicated the importance of providing these subjects with support through systematized interventions and in the long term, emphasizing that individual readiness to change may not be sufficient. It is therefore necessary to intervene directly through offering activities which promote food education, healthy habits, and the regular practice of physical activity^17^.

In Brazil, a study with 145 users of the health service, which used TM for assessing the eating-related behavior, observed that individuals classified in the initial stages, such as "contemplation" and "precontemplation" were more susceptible to having a diet rich in fats. Those, on the other hand, who were in a stage of "action" showed greater concern with the quality of their eating habits^18^. This condition, therefore, can be determinant for the individual who has as her proposal to begin a process of behavioral change with the aim of losing weight.

In analyzing specifically the issues which are integral to each one of the domains of the SRBC, one can observe that habits related to adjustment of eating habits determined by quantity of food, consumption of fruits and vegetables and quantity of fat were significantly changed for the women of the IG. It is emphasized that food choice is a complex process which involves numerous determinants, such as experiences acquired throughout life, influences of cultural ideals, personal factors, resources available and demographic determinants, among others^19^ ^-^ ^20^ ^).^.

One factor which is related directly to success in behavioral change is the perception of the risk that excess weight can cause in health. One Brazilian study which assessed behavioral change related exclusively to consumption of fruits and vegetables indicated that 83.3% of the individuals who were most concerned with appropriate consumption were in the stages of action and maintenance^21^ ^).^

Another study which evaluated whether the SRBC are related to consumption of fat evidenced that all the 131 women evaluated consumed more fat than recommended, and that the consumption of fat was lower among the women who were in the stages of action and maintenance^22^. The authors emphasized that the use of the questionnaire for classification of the SRBC makes it possible to identify the eating-related errors present and, as a consequence, makes it possible to promote a direct and accurate intervention regarding these^22^.

It is highlighted that, in recent decades, how Brazilians eat has changed in ways that have been reflected directly in their body composition. One frequently-observed change is the increase in the consumption of ultra-processed foods, it being common for these to contribute more than half of the total calories consumed. As a result, greater consumption of ultra-processed foods is associated positively with the excessive consumption of harmful substances such as fats, cholesterol, sodium and calories - and negatively, with the consumption of carbohydrates, proteins and dietary fiber, which triggers weight gain and metabolic changes^23^.

As a result, it is believed that nutritional guidance favors the development of individual strategies which can influence the choice of foods, as with the quantity and quality of what is consumed.

In spite of the low quality of evidence related to the impact of the interventions regarding sustainable behavioral change, even with multi-professional actions, it is observed that the individuals evaluated through this model tends to seek improvements in the quality of their food, principally reducing consumption of fat and increasing consumption of fruits and vegetables^21^, as observed in the present study. One systematic review on interventions based in theoretical models indicates, with a strong degree of evidence, that individuals benefit from the diet-related changes implemented^24^.

As well as a balanced diet, the habitual practice of physical activity is a fact which contributes, both to weight loss and to the maintenance of body weight^25^. The domain corresponding to the active behaviors presented significant results for the IG after the intervention, with emphasis on the issues related to daily habits. Based in the results presented, it is highlighted that the women of the IG presented significant changes regarding the inclusion of daily habits of physical exercise. 

It is emphasized that there is a shortage of studies, particularly in the Brazilian ambit, addressing the impact of interventions on changing behaviors and lifestyle. This is probably related to practical difficulties in holding this type of study, such as the constitution of homogenous groups and the impossibility of controlling the dropping-out of volunteers over the course of the studies. 

The limits of this study's results relate to the small number of participants, which made it impossible to generalize from its results; to the possibility of contamination between the groups, as the study was undertaken in a small municipality, it being probable that at least some of the participants knew each other; and to the fact that the participants of the IG had frequent contact with health professionals, which may have influenced the results of this group (the Hawthorne effect). 

In spite of these limitations, this study presents relevant contributions for the advancing of knowledge in the area, in that its results suggest that interventions which incorporate diet-related guidance, allied with encouragement to the practice of physical activity in a group, may help to prevent the increase in rates of obesity in women. They also indicate the need for further studies for a better understanding of the state of readiness for action in behavioral change among women with overweight and obesity; and likewise for studies which involve a larger sample and in which there is greater control of the variables related to the intervention in the members of the control group. 

## Conclusion

The study's results show that, generally speaking, the participants from the two groups were predisposed to undertaking behavioral changes; however, only the women from the IG presented anthropometric indicators that the changes had really taken place. 

The results allow one to infer that for significant behavioral changes to happen, that is, for there to be impact on the anthropometric components, it is not enough for the individuals to present good indicators for disposition for change. It is necessary for there to be support, encouragement and the offering of actions which aim to promote the regular practice of physical exercise and a balanced diet. It is emphasized that in this study in particular, the multi-professional support was important for the changes observed to occur. 
